# Virtual reality delivered exposure for fear of needles: a small-scale pilot

**DOI:** 10.3389/fpsyt.2025.1642988

**Published:** 2025-09-11

**Authors:** Anna Caltabiano, Taylor Burke, Jacqueline Nesi, Martina Di Simplicio, Nejra van Zalk

**Affiliations:** ^1^ Dyson School of Design Engineering, Imperial College London, London, United Kingdom; ^2^ Department of Psychiatry, Harvard Medical School/Massachusetts General Hospital, Boston, MA, United States; ^3^ Department of Psychiatry, Warren Alpert Medical School of Brown University, Providence, RI, United States; ^4^ Division of Psychiatry, Department of Brain Sciences, Imperial College London, London, United Kingdom

**Keywords:** fear of needles, anxiety, virtual reality, exposure, VR

## Abstract

**Background:**

Fear of needles significantly impacts individual and public health by leading many adults to avoid necessary medical procedures, including vaccinations and blood tests. Virtual Reality Exposure-Based Therapy has shown promise as an effective and accessible intervention for anxiety disorders but remains under-explored.

**Objectives:**

This study aimed to evaluate the efficacy and acceptability of a single-session virtual reality intervention targeting fear of needles in adults.

**Methods:**

A total of 62 adults reporting needle fear were recruited into experimental (n = 32) and online comparison groups (n = 30). The experimental group completed one Virtual Reality Exposure-Based Therapy session, which comprised of two self-paced virtual reality exposures simulating medical needle procedures. Anxiety and affect were assessed at baseline, during, and immediately following virtual reality exposures, and at a one-month follow-up. Acceptability, usability, presence, plausibility, and virtual reality sickness were also measured.

**Results:**

The intervention successfully elicited anxiety during exposure. At one-month follow-up, a modest but statistically significant reduction in symptom severity was observed on one measure (Specific Phobia Questionnaire), though no significant change was noted in life interference or on another severity measure (Medical Fears Survey). Participants rated the intervention highly in terms of usability and acceptability, although some reported symptoms of virtual reality sickness (e.g., disorientation, motion sickness).

**Conclusions:**

Virtual Reality Exposure-Based Therapy appears to be an effective and highly acceptable intervention for reducing immediate anxiety related to needle exposure, demonstrating strong potential as a scalable, accessible alternative to traditional exposure therapy. However, further research is necessary to confirm these findings, optimize intervention protocols, and examine long-term effectiveness for fear of needles.

## Introduction

Fear of needles, also known as *needle phobia* or *trypanophobia*, is characterized by an intense fear of medical procedures involving needles or injections. It often manifests with physiological symptoms such as increased heart rate, a sudden drop in blood pressure leading to fainting, or feelings of panic when exposed to needles (1). While fear of needles is widely recognized, it remains underdiagnosed and undertreated in the adult population, leading many to avoid necessary medical procedures due to embarrassment or distress (1).

The documented prevalence of fear of needles is substantial, affecting approximately 20-30% of adults, with higher rates in younger adults and women (1). The prevalence is even higher among individuals with chronic illnesses, with rates varying widely from 0.2-80% in diabetes patients, 17–52% in cancer patients, and 25–47% in those with chronic kidney disease (2). The avoidance behaviors associated with fear of needles can have significant public health implications, particularly when they interfere with vaccination adherence, blood donation, and routine medical care, contributing to poorer health outcomes.

Despite the medical and public health burden, fear of needles is rarely formally diagnosed or treated (3). While prior reviews have identified a number of studies on this topic (1), relatively few have focused specifically on general adult populations. Current first-line treatments include applied tension techniques to prevent fainting, and breathing exercises to manage panic responses (1). However, these methods do not address the underlying fear and avoidance behavior that perpetuate the phobia (4). Another first-line intervention, exposure-based therapy, directly targets these underlying mechanisms. While applied tension can be a useful adjunct to prevent vasovagal syncope during exposure, there is little evidence that it enhances the efficacy of exposure therapy in reducing fear itself (4).

### Exposure-based therapy

Exposure therapy is the gold-standard treatment for anxiety-related disorders, including specific phobias (5). It involves gradual, systematic exposure to the feared object or situation, helping individuals learn that their feared outcomes do not materialize, ultimately reducing their anxiety response (6, 7).

Exposure therapy is grounded in several theoretical models, primarily habituation and inhibitory learning. Traditionally, the habituation model posits that repeated exposure to a feared stimulus leads to a reduction in anxiety through a natural decline in the fear response over time (8). However, more contemporary frameworks, such as the inhibitory learning model, suggest that exposure works by fostering new, non-threatening associations that inhibit the original fear memory rather than erasing it (9).

The exposure approach is commonly incorporated into cognitive-behavioral therapy and can be delivered in single- or multi-session formats. Recent research indicates no significant difference in efficacy between the two; however, a single session exposure protocol is more time-efficient (6). Despite its effectiveness, traditional exposure therapy can be costly and requires access to trained clinicians, which may not be feasible for many individuals seeking treatment (10).

### Virtual reality-delivered exposure-based therapy

Virtual Reality Exposure-Based Therapy (VRET) is an innovative approach that offers an immersive and controlled environment for exposure-based treatment using virtual reality (VR) to deliver exposure. VRET has been found to be effective in treating various anxiety disorders, including post-traumatic stress disorder, social anxiety disorder, and specific phobias (11, 12). By providing a virtual environment that simulates real-life scenarios, VRET eliminates many of the barriers associated with traditional exposure therapy, such as the need for in-person exposure to feared stimuli and access to specialized clinicians.

Despite the success of VRET in treating various anxiety-related disorders, its application to fear of needles remains under-explored. Many studies that utilize VR technology as a possible intervention for fear of needles investigate its usage as a distraction mechanism (e.g., 13–15), rather than a vehicle for exposure. Additionally, many studies on needle fear (e.g., 16, 17) focus on specific populations–such as children aged 5–12 and adults with Autism Spectrum Disorder–who are more prone to heightened fear responses and avoidance. In these cases, avoidance may be so significant that it necessitates more intensive interventions, such as physical restraint or sedation, particularly when individuals do not fully comprehend the importance of medical procedures.

To date, there is only one study (18) that investigates the use of modern, non-smartphone-based head-mounted display VRET as an intervention for fear of needles in adults. This study was a pilot trial of 43 participants randomized into a single-session of VRET or a wait-list control group (18). A single-session of VRET demonstrated the potential to alleviate fear of needle-related symptoms (18). However, the study included solely diagnosed individuals, leaving a significant gap in the literature regarding the use of VRET for adults along a fear of needles spectrum. Another study (19) employed smartphone-based VR delivered using 360° video and included participants with various phobias, including fear of needles. However, this approach differs in both technological immersion and the level of user interaction.

In addition, while existing studies often focus on symptom reduction, they frequently neglect the user experience of the intervention. This omission is especially critical, given that factors such as presence, plausibility, VR sickness, and usability can influence both participant engagement and therapeutic outcomes. In VRET, the user’s perception of the environment and comfort with the technology may be just as important as the therapeutic content itself. Furthermore, even the most effective intervention will fail if it is not deemed acceptable by those who need it; individuals are unlikely to engage with a treatment they find confusing, uncomfortable, or unpleasant. Therefore, examining user experience is essential to understanding not just whether VRET works, but for whom and under what conditions it is most effective.

### This study

The current study aims to address the need for more research on VRET for fear of needles and to build on the findings of 18 by evaluating the efficacy and acceptability of a VRET intervention for fear of needles in adults. Specifically, the study seeks to answer the following research questions pertaining to intervention efficacy and user experience:

#### 1) Efficacy of the intervention

Does the intervention elicit anxiety as would be expected in a standard exposure-based protocol?

How effective is VRET in reducing fear of needles symptoms?

#### 2) User experience and acceptability

Do participants find the intervention acceptable?

How do participants perceive the VR experience (including VR sickness)? Based on participant feedback, what aspects of the intervention could be improved to enhance user experience and therapeutic outcomes?

As an additional exploratory investigation, the study also seeks to answer whether individuals who underwent a single session VRET comprising of two exposures experienced reduced levels of anxiety when subsequently encountering images of needles and injections immediately after the intervention, when compared to individuals who did not experience the VRET.

By exploring these research questions, this study seeks to advance the understanding of VRET as a promising intervention for fear of needles and to explore its potential for improving scalable and accessible treatment options for affected individuals.

## Methods

### Participants

Two groups of participants were recruited: an experimental group and an online comparison group. Participants in the experimental group were recruited via Instagram advertisements and posters placed around the main campus of Imperial College London. The advertisements and posters directed participants to an online screening survey on Qualtrics. Inclusion criteria were being 18 years or older, English speaking, and reporting a fear of needles. The latter was defined as scoring a 1 or higher on the Specific Phobia Questionnaire (SPQ) blood-injection-injury subscale and/or scoring a 1 or higher on the Medical Fears Survey (MFS) injections and blood subscale.

Exclusion criteria were reporting any condition precluding adherence to the measurement protocol (such as difficulty reading or writing), any uncorrected visual and/or auditory impairment affecting full participation in VR (use of corrective lenses or hearing aids was not a criterion for exclusion), current or past diagnosis of fear of needles, current or past treatment for fear of needles (excluding self-help), self-reported history of fainting from the sight of blood, presence or history of epilepsy, and history of severe motion sickness or dizziness. Individuals were also excluded if they self-reported currently taking medication that affected heart rate, such as benzodiazepines, as this could artificially decrease anxiety responses. Participants with a current or past diagnosis or treatment for fear of needles were excluded to ensure a treatment-naive sample and reduce potential confounding from prior clinical exposure or intervention. Those with a history of fainting at the sight of blood were excluded primarily for safety reasons, based on ethics committee guidance, and secondarily to avoid conflating fear of blood with fear of needles, given their potentially distinct psychological and physiological profiles.

Participants in the online comparison group were recruited via Prolific. They had the same inclusion and exclusion criteria as the experimental group, with the added criterion of self-reporting as UK-based and responding “yes” to “Would you be willing to participate in trialing a virtual reality intervention for fear of needles?”. This inclusion criterion was established to enhance the comparability with the experimental group. They were also asked to respond to two attention-check questions and were only included if they answered correctly to both, ensuring they were reading and responding to the survey carefully. Due to resource and planning constraints, comparison group participants did not complete the one-month follow-up survey. As this was an exploratory aspect of the study, we acknowledge this as a limitation for between-group longitudinal comparisons.

Previous VRET studies reported effect sizes ranging from 0.88 to 1.08 (11, 12). With an alpha of 0.05 and a power of 0.95, a sample size of 37 participants in each group is required to detect a *d* = 0.88 effect size, and 25 participants in each group are required to detect a *d* = 1.08 effect size. Given the *a priori* power analysis, feasibility of recruitment, and limited research on effect size for this topic, we aimed to recruit 25 to 37 participants per group.

A total of 62 participants (32 in the experimental group and 30 in the comparison group) were recruited and provided informed consent. All participants in the experimental group who completed the in-lab session received £7 reimbursement as an Amazon voucher. If they also completed the one-month follow-up survey, they received an additional £3. All 32 participants in the experimental group completed the in-lab portion of the study. Due to a fire alarm during one participant’s session, data from the onset of the alarm to session end was partially excluded and subsequently imputed to retain as much data as possible. 2 participants did not complete the follow-up survey. Participants in the comparison group were reimbursed £10/hour through Prolific. 75 potential participants responded to the Prolific advertisement and met the inclusion and exclusion criteria. Of these, 30 participants completed the comparison group surveys and correctly answered both attention check questions, constituting the final comparison group.

All study procedures were conducted in accordance with the ethical principles outlined in the World Medical Association’s Declaration of Helsinki. Ethics approval for study measures and protocol was granted by the Research Governance and Integrity Team at Imperial College London (ref: 6577424).

### Procedure

Participants in the experimental group who met the inclusion criteria and consented to take part in the study were invited to an in-lab session where they were given an online Qualtrics survey to collect baseline information on fear of needles symptoms using the Specific Phobia Questionnaire (SPQ; 20) and the Medical Fears Survey (MFS; 21) subscales and trait anxiety using the Patient Reported Outcomes Measurement Information System - Anxiety (PROMIS anxiety; 22). Participants were then given a brief information sheet about exposure therapy and read a short script providing the context to the VR scenario they would experience shortly. Before entering the VR environment, participants were asked to select the skin color of their VR avatar. This was done to enhance the experience of embodiment in the virtual environment. Participants then experienced two identical self-paced VR exposures and viewed a short neutral video between exposures to return to baseline. The general single-session VRET approach is consistent with prior protocols (e.g., 18, 23, 24). However, the single session was split into two identical exposures to reduce novelty-induced anxiety during the second exposure. The five-minute neutral video, which showed clips of animals from the documentary series *Baby Planet*, has been used in previous psychological studies to reset affective responses between tasks. Each exposure was self-paced, meaning participants were able to move through the VR scenario at their own pace and indicate to the researcher when they were ready to proceed through each stage. On average, each exposure lasted approximately ten minutes.

This duration reflected the finite sequence of actions and events available in the VR software, which included the following in order: overhear other avatars discuss their recent injection experiences, entering the clinic room from the waiting room, experiencing a finger-prick blood test, an injection, a blood draw, and finally a longer (i.e., multi-vial) blood draw. During each exposure, anxiety was measured four times using the Subjective Units of Distress Scale (SUDS; 25), administered verbally. The Positive and Negative Affect Schedule shortform (PANAS; 26) was administered three times: after the first exposure, after the break between sessions, and after the second exposure.

When participants were virtually pricked with a needle during the VR exposures, the sensory experience was replicated in reality by the experimenter poking the participant in the same location on the same arm with a blunt tool. This was done to further enhance embodiment during the VRET intervention.

Following the second VR exposure, participants completed several post-intervention measures. These included the Simulator Sickness Questionnaire (SSQ; 27), a set of custom questions assessing VR presence and plausibility adapted from Slater (28), and the System Usability Scale (SUS; 29). Participants also responded to open-ended questions about the acceptability and usability of the VR experience (e.g., “How was your experience and the way you felt similar to your experience receiving a real injection? How was it different?” and “In your opinion, is there anything that could have been done differently to improve the software or create a better experience in any aspect?”).

Immediately after completing these measures, participants viewed three photos of needles and injections, and were asked about their levels of anxiety (SUDS) immediately before and after. Positive and negative affect (PANAS) was also measured after viewing the photos. The PANAS administered after the second exposure also served as the pre-photo affect measure. These photos were selected for ability to elicit anxiety in a previous focus group of individuals with heightened fear of needles and injections.

**Figure 1:**
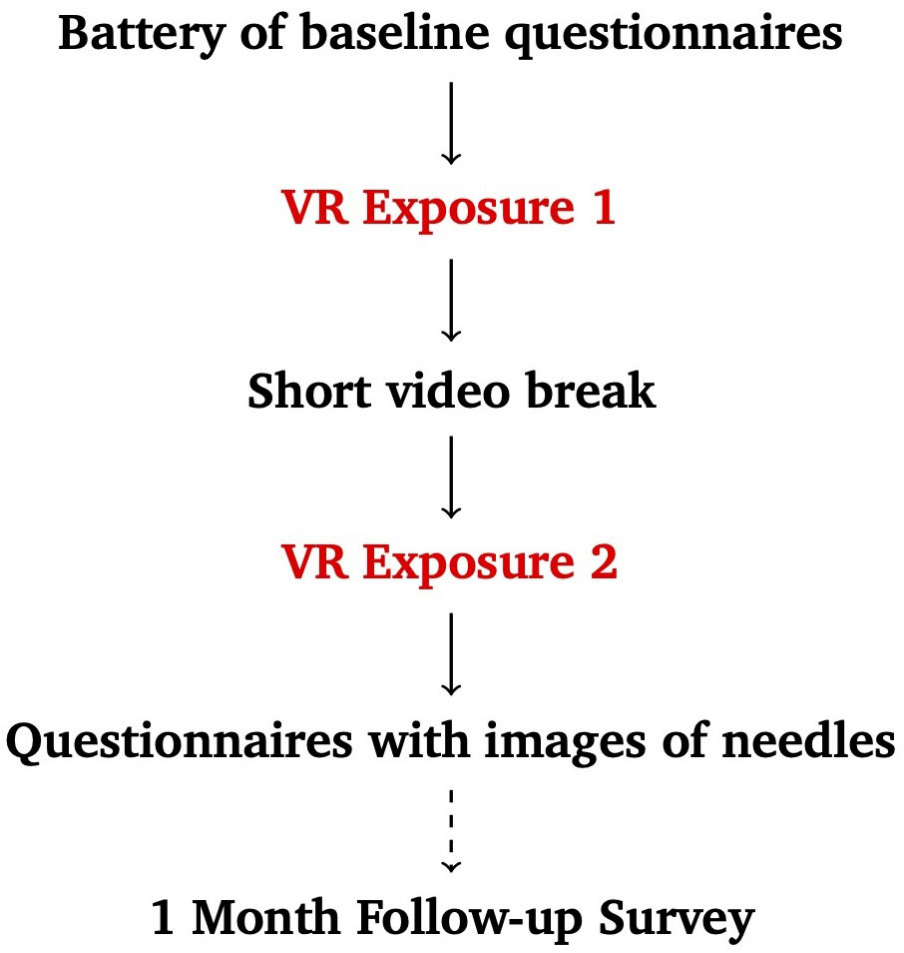
Experimental group protocol.

One month later, an online follow-up survey was emailed to participants. They first responded to open-ended questions about their attitudes relating to needles. They then completed the SPQ and MFS subscales to assess fear of needles symptoms and associated life interference, as well as the SUDS for momentary anxiety. After viewing the same three needle images, they again completed the SUDS and PANAS to assess post-photo anxiety and affect. A schematic of the experimental group’s protocol is shown in **Figure 1**.

Participants in the comparison group who met the inclusion criteria and gave consent were directed to an online Qualtrics survey. The survey collected information on fear of needles symptoms (MFS and SPQ), life interference (SPQ), baseline positive and negative affect (PANAS), and baseline anxiety (PROMIS anxiety). They were then asked to view the same needle and injection photos as the experimental group and completed the SUDS and PANAS immediately before and after. The comparison group did not receive the VRET intervention.

### Measures

#### Demographics

Participants self-reported age, sex, and if gender identity was the same as sex registered at birth.

#### Psychiatric diagnoses

Participants self-reported any past or current psychiatric diagnoses.

#### Fear of needles and injections

Participants reported symptoms concerning fear of needles and injections using the blood-injection-injury subscale of the symptom severity Specific Phobia Questionnaire (SPQ) at baseline (*α* = 0.74) and at one-month follow-up (*α* = 0.85), as well as the injections and blood subscale of the Medical Fears Survey (MFS) (baseline: *α* = 0.83, follow-up: *α* = 0.80). The SPQ was also used to measure life interference at baseline (*α* = 0.76) and at one-month follow-up (*α* = 0.76). The SPQ subscale (20) consists of two 14-item scales (one measuring symptom severity and one measuring life interference of these symptoms) on a 5-point Likert scale, ranging from 0 (no fear/no interference) to 4 (extreme fear/extreme interference). The MFS subscale (21) consists of 4 items on a 4-point Likert scale, ranging from 0 (no fear or concern at all), 1 (mild fear), 2 (considerable fear), to 3 (intense fear). Open-ended questions were also included to provide descriptive information about participants’ attitudes toward needles and injections.

Clinical cutoff scores for the SPQ and MFS are not universally established. Receiver Operating Characteristic analyses have been used in prior research to identify sample-specific thresholds on the SPQ symptom severity subscale, with scores ≥ 20 often indicating higher symptom severity (20). However, these thresholds are not validated as formal diagnostic cutoffs. No similar sensitivity or specificity indicators exist for the MFS subscale. Consequently, scores in the current study were interpreted cautiously and used primarily for within-sample comparison rather than formal diagnosis.

#### Anxiety & affect

Baseline trait anxiety was measured using Patient Reported Outcomes Measurement Information System - Anxiety (PROMIS anxiety), a 29item questionnaire with a 5-point Likert scale (*α* = 0.96; 22). Response items ranged from 1 (never) to 5 (always). Momentary (or state) anxiety was measured using Subjective Units of Distress Scale (SUDS), which is one item with an 11-point Likert scale (responses ranging from 0 to 10) and was verbally administered during each VR exposure (25). To measure positive and negative affect, the short-form of the Positive and Negative Affect Schedule (PANAS-SF; 30) was administered at baseline (*α* = 0.80), after the first VR exposure (*α* = 0.93), after the break between exposure sessions (*α* = 0.84), and after the second VR exposure (*α* = 0.89). It was also given in the follow-up survey (*α* = 0.94). The PANAS comprised two scales (one for positive affect and one for negative affect), both on a 5-point Likert scale, from 1 (very slightly or not at all) to 5 (extremely). The PANAS-SF was administered in its state format, where participants rated how they felt “right now” rather than over the past week. While the PANAS-SF has not been formally validated for state measurement, it has been used in prior studies to assess momentary affective responses to brief interventions (e.g., 31). Importantly, the original PANAS has demonstrated strong psychometric properties for both trait and state affect measurement, providing a conceptual foundation for this adaptation (30). Measuring state affect across time points enabled us to examine the emotional impact of the intervention, including whether participants experienced emotional relief, regulation, or recovery following virtual exposure. Prior VRET studies have documented reductions in negative affect and increases in positive affect following virtual exposure sessions (e.g., 32, 33). Reductions in negative affect following exposure therapy may indicate habituation or reduced fear generalization, while increases in positive affect may reflect perceived coping or mastery (34, 35). These dynamics help contextualize the immediate psychological impact of a brief, single-session exposure.

#### User experience & acceptability

VR presence and plausibility were measured by two questions modeled closely after Slater (28). To measure plausibility, we asked participants to compare the virtual environment to receiving 1) a real injection and 2) an imagined injection. The responses are on a 5-point Likert scale, where 1 is much more anxious in VR and 5 is much more anxious in the real or imagined injection. Responses to each item were analyzed individually. VR sickness was measured using the Simulator Sickness Questionnaire (SSQ) at baseline (*α* = 0.81) and post-intervention (*α* = 0.83). The SSQ is on a 4-point Likert scale, ranging from 0 (not at all) to 3 (severe) (27). To assess usability, participants completed the System Usability Scale (SUS), which has a 5-point Likert scale from 1 (strongly disagree) to 5 (strongly agree) and an alpha of 0.88 (29). Scores range from 0 to 100 with a score of ≥ 68 being indicative of above-average user experience (36). It is a validated questionnaire widely used to evaluate user experience with digital systems (37). While the SUS does not provide a direct psychometric assessment of acceptability, we included it as a pragmatic, quantifiable proxy for usability-related components of acceptability. This approach is consistent with recent digital health research (e.g., Bangor et al. (36)Collins-Pisano et al. (38), and Isaacs et al. (39)) and is conceptually aligned with the Theoretical Framework of Acceptability (40), which identifies usability and burden as key constructs.

To capture a broader understanding of acceptability beyond usability, we supplemented the SUS with open-ended questions for descriptive analysis, addressing experiential, cognitive, and affective dimensions of the intervention. These included prompts such as: “How did you feel while you were taking part in the virtual reality experience?” and “How was your experience and the way you felt similar to your experience receiving a real injection? How was it different?” These items allowed us to assess subjective impressions of the intervention’s realism, emotional impact, and overall acceptability from the user’s perspective.

### Equipment & environment

**Figure 2:**
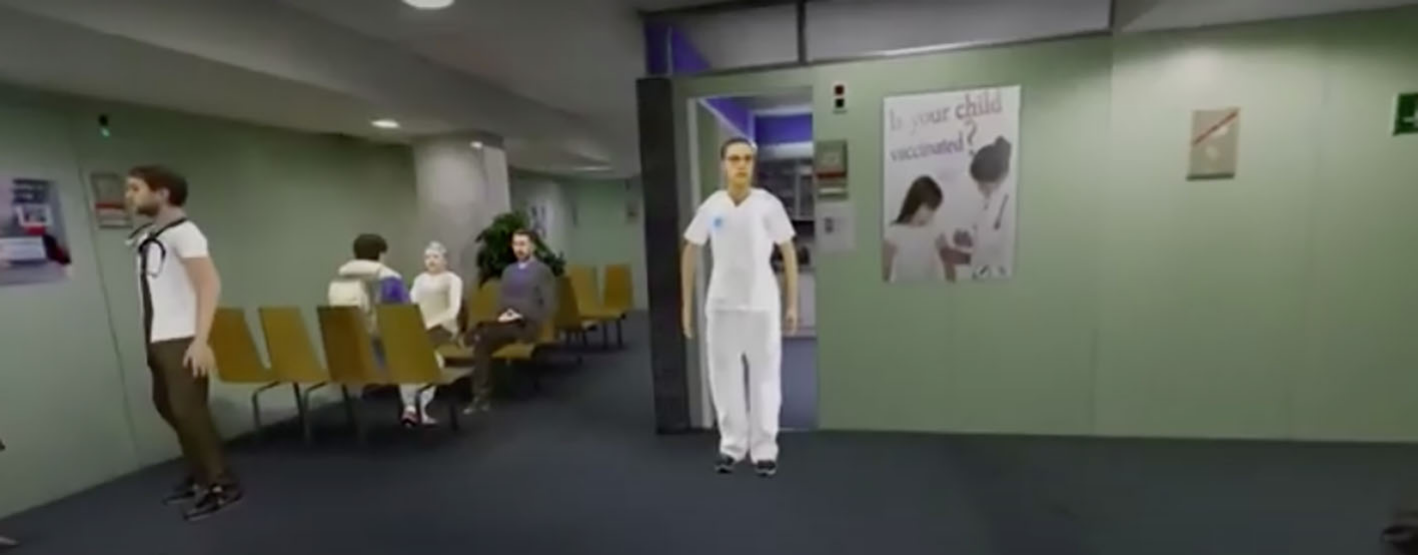
Screenshot of the VR environment depicting a medical clinic.

The VR environment we used was developed by Amelia by XRHealth. The participants engaged in an environment depicting a medical clinic (**Figure 2**).

**Figure 3:**
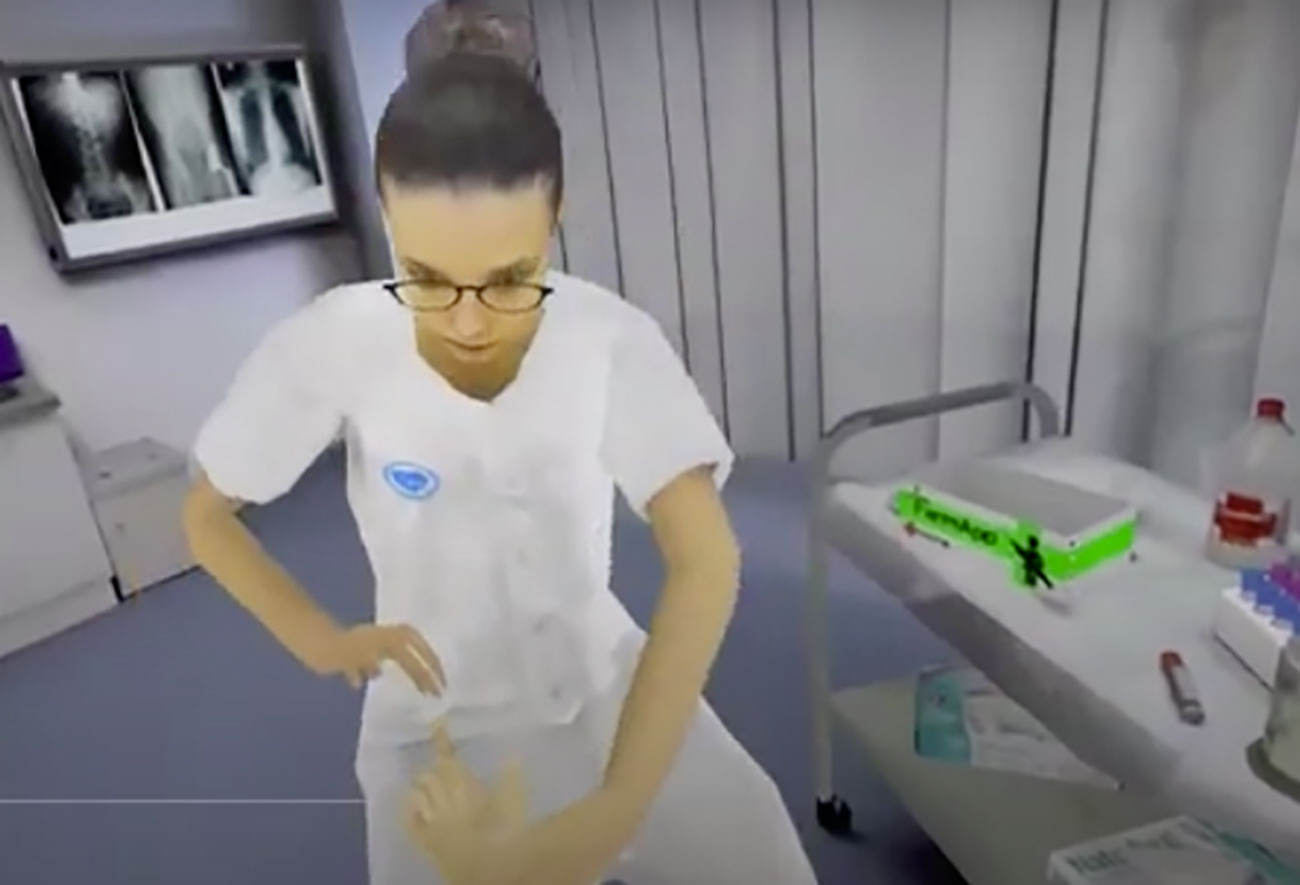
Screenshot of the VR environment depicting a finger-prick blood test.

Participants began sessions by sitting in a clinic waiting room, where other patients can be heard discussing their recent injections and characterizing them as painful. A clinician then invited the participant into the clinic room. Upon entering, they saw vials and other blood-taking equipment as the clinician prepared for the appointment. The participant sat in the patient chair (i.e., they simultaneously sat in a chair in the real world to mirror their position in VR) and experienced a virtual finger-prick blood test, injection, blood draw, and a longer (i.e., multi-vial) blood draw (**Figure 3**). The software was run on a PICO G2 head-mounted display (HMD) VR headset.

The VR environment was controlled through Amelia by XRHealth’s online platform. In this study, participants completed the actions in the given order (i.e., overhear other avatars discuss their recent injection experiences, enter the clinic room from the waiting room, finger-prick blood test, injection, blood draw, and longer blood draw) at their own pace. To limit VR sickness and heighten the sense of plausibility and immersiveness of the virtual environment, participants were asked to remain seated during the VR session.

### Data analysis

This study was preregistered (https://aspredicted.org/ykc7-62nr.pdf) with several deviations from the original preregistration. Specifically, the Distress Tolerance Scale and the Patient-Reported Outcomes Measurement Information System Anxiety & Perceived Stress measures were not included in the current analyses, as they were beyond the scope of the current research objectives but may be examined in future analyses. Additionally, physiological data collected during the study were not reported in this paper, but will be reported and analyzed in a subsequent paper. Sample size adjustments were also made, resulting in 32 participants per group instead of the initially planned 30 participants per group, to accommodate recruitment outcomes. Furthermore, the originally intended Bayesian analyses were replaced by frequentist analyses, as frequentist methods provided greater alignment with the exploratory nature of the data and facilitated interpretation of results within the existing research framework. Lastly, descriptive analyses of open-ended questions using Linguistic Inquiry and Word Count software were omitted in favor of descriptive reporting to streamline the presentation and discussion of results.

Statistical analyses were performed in R 4.4.0. Data were analyzed to explore efficacy of the intervention in evoking anxiety, efficacy in reducing symptoms, and user experience. To examine efficacy of the intervention in evoking anxiety and explore changes in positive and negative affect, Wilcoxon rank-sum tests and Friedman Rank Sum tests were used to compare pre- and post-intervention scores for negative affect, positive affect, and anxiety levels. Paired t-tests were used to examine efficacy of the intervention in reducing symptoms of fear of needles. Effect size calculations were conducted to investigate magnitude of changes. To further evaluate the possible effects of a single session, Wilcoxon rank-sum tests were performed to compare participants’ anxiety levels and related magnitudes of change after viewing photos of needles and injections. To examine user experience, descriptive data for participants’ virtual sense of presence and plausibility, descriptive data for intervention acceptability, frequency of item endorsement on the SUS, and open-ended participant feedback for areas of intervention improvement were investigated. Exploratory analysis using Wilcoxon Signed Rank tests comparing the experimental and comparison groups examined if the VRET intervention elicited anxiety. To further explore the potential effects of a single session of VRET on fear of needles, participants’ positive and negative affect were compared between groups after they had encountered images of needles and injections using Wilcoxon rank-sum tests.

2.15% of the experimental group’s data and 0.91% of the comparison group’s data were missing either at Random or Completely at Random. This was addressed using random forest run via the MissForest R package, which outperforms comparable data imputation methods (41). Statistical significance was defined at p <.05.

## Results

The experimental group comprised 25 females and 7 males aged 18-48 (*M* = 25.31, *Mdn* = 21, *SD* = 8.60). 96.88% identified their gender as the same as the sex they were assigned at birth. 13 participants in the experimental group reported having prior VR experience. The comparison group comprised 18 females and 11 males, with 1 participant preferring not to say, aged 22-74 (*M* = 36.90, *Mdn* = 37, *SD* = 11.80). 96.77% reported that their gender aligns with the sex assigned to them at birth.

The participants’ baseline fear of needles symptoms, as measured by the SPQ and MFS subscales, as well as life interference due to fears are displayed in **Table 1**.

**Table 1:** Baseline fear of needles and associated life interference.

Measure	Experimental group			Comparison group			Wilcoxon rank-sum test	
	M	Mdn	SD	M	Mdn	SD	W	P
Symptom Severity (MFS)	10.03	10.00	3.23	6.66	6.0	2.65	767.50	<.001
Life Interference (SPQ)	29.31	27.00	11.22	24.07	20.00	10.76	638.50	0.03
Symptom Severity (SPQ)	41.06	43.00	9.60	30.07	27.50	12.02	740.00	<.001

This table represents data pre-imputation.

A table of correlations between the main study variables are displayed in **Table 2**.

**Table 2:** Correlation table of main study variables.

Variable 1	Variable 2	Correlation	FDR-corrected p-value
baseline life interference	age	0.03	0.90
baseline severity (SPQ)	age	-0.05	0.83
baseline severity (MFS)	age	-0.26	0.13
baseline pos affect	age	0.29	0.08
baseline neg affect	age	-0.02	0.93
PROMIS anxiety	age	-0.03	0.90
anxiety in exposure 1	age	0.13	0.71
anxiety in exposure 2	age	0.12	0.73
life interference after VRET	age	0.30	0.21
severity after VRET (SPQ)	age	0.28	0.23
severity after VRET (MFS)	age	-0.04	0.90
baseline severity (SPQ)	baseline life interference	0.80	*<.*001
baseline severity (MFS)	baseline life interference	0.73	*<.*001
baseline pos affect	baseline life interference	0.10	0.68
baseline neg affect	baseline life interference	0.24	0.17
PROMIS anxiety	baseline life interference	0.28	0.10
anxiety in exposure 1	baseline life interference	0.19	0.46
anxiety in exposure 2	baseline life interference	0.32	0.17
life interference after VRET	baseline life interference	0.56	0.01
severity after VRET (SPQ)	baseline life interference	0.47	0.03
severity after VRET (MFS)	baseline life interference	0.44	0.05
baseline severity (MFS)	baseline severity (SPQ)	0.80	*<.*001
baseline pos affect	baseline severity (SPQ)	0.04	0.90
baseline neg affect	baseline severity (SPQ)	0.18	0.31
PROMIS anxiety	baseline severity (SPQ)	0.26	0.12
anxiety in exposure 1	baseline severity (SPQ)	0.23	0.35

### Efficacy of the intervention

**Figure 4:**
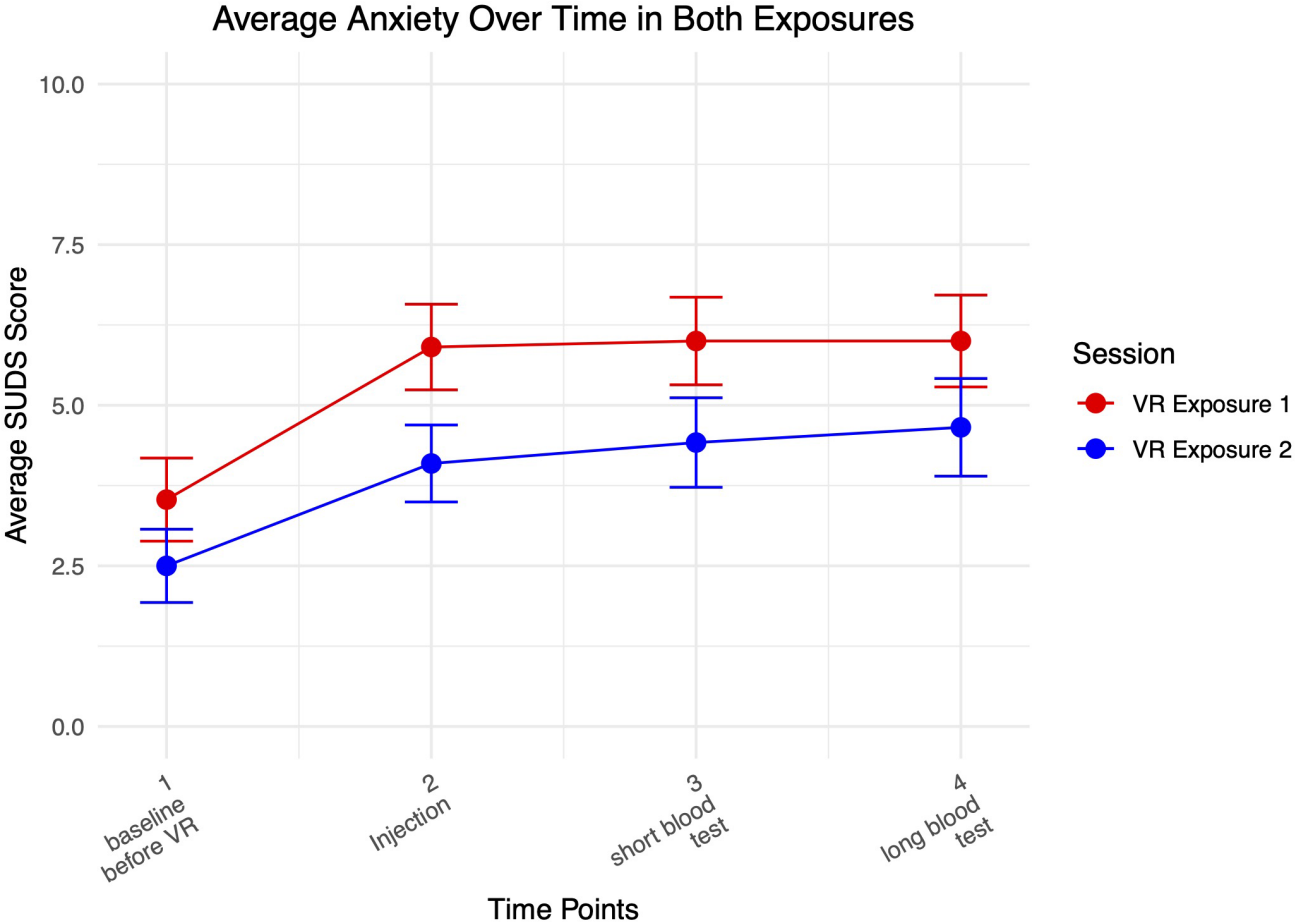
Anxiety during both VR exposures.

**Figure 4** shows a graph of average anxiety levels over both VR exposure sessions.

Anxiety increased from baseline as participants interacted with the medical clinic VR environment and received virtual injections, and remained elevated as they received blood tests. Highest levels of anxiety were achieved when interacting most with the virtual needles. Participants reported higher average levels of anxiety during the first compared to the second exposure.

**Figure 5:**
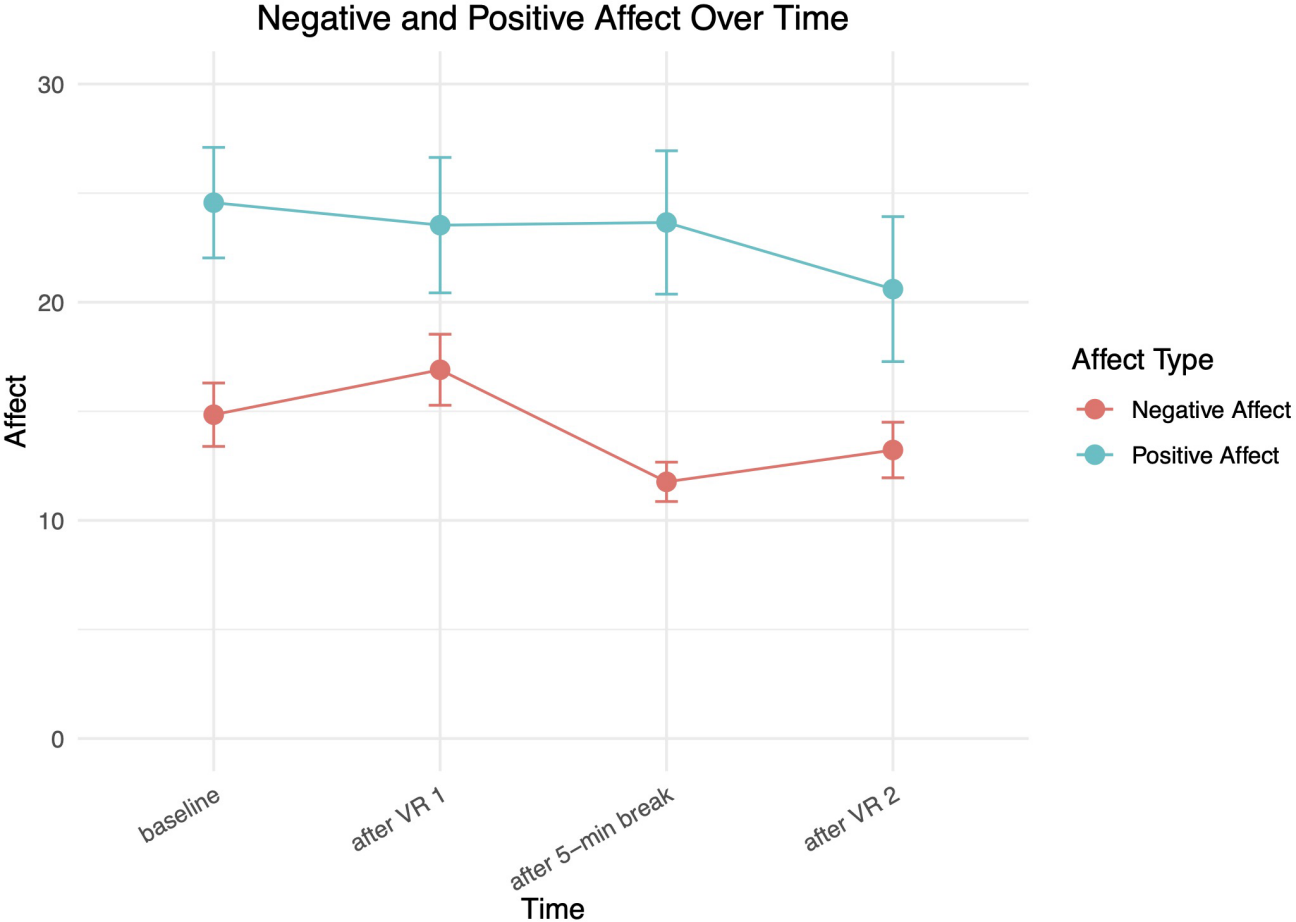
Average positive and negative affect across both VR exposures.

**Figure 5** shows average negative and positive affect over time, with positive affect remaining relatively level and then dropping slightly after the 2nd VR exposure. Negative affect increased slightly from baseline to after the 1st VR exposure and decreased as expected during the break, before increasing slightly again during the 2nd VR exposure.

To examine if the VRET intervention elicited anxiety and how it affected positive and negative affect in the experimental group, a series of Wilcoxon Signed Rank tests were conducted (**Table 3**). A statistically significant reduction in anxiety was demonstrated in the experimental group from baseline to post-intervention (*p* = .02). A statistically significant change in positive affect was also found (*p <* 0.001). As expected, no significant change was seen in negative or positive affect in the comparison group, who did not receive the intervention.

**Table 3:** Wilcoxon Signed Rank test results at baseline versus post-intervention.

Measure	V	p-value	Median difference
Experimental Group			
Anxiety (SUDS)	329.00	0.02	-1.00
Negative affect (PANAS)	171.50	0.48	0.04
Positive affect (PANAS)	400.00	*<.*001	-6.30
Comparison Group			
Negative affect (PANAS)	77.50	0.19	0.00
Positive affect (PANAS)	214.00	0.07	-0.50

To investigate changes in affect and anxiety during the intervention, in accordance with the assumptions of normality, Friedman tests were conducted for the repeated measures (**Table 4**). Statistically significant change (*p <* 0.001) was seen for positive affect, negative affect, and anxiety during the VRET.

**Table 4:** Friedman test results for anxiety and affect during intervention.

Measure	Chi-Squared (*χ* ^2^)	df	p-value
Neg affect (PANAS)	40.07	3	*<.*001
Pos affect (PANAS)	34.67	3	*<.*001
Anxiety (SUDS) during exposure 1	38.51	3	*<.*001
Anxiety (SUDS) during exposure 2	34.94	3	*<.*001

To examine the efficacy of a single session of VRET in reducing fear of needles symptoms, a paired t-test was conducted on symptom severity score at baseline pre-intervention and at one-month follow-up using the MFS. The results were not statistically significant (*t*(31) = 1.39, *p* = .18, 95% CI [-0.30, 1.57]). A paired t-test was also conducted using SPQ as a measure of symptom severity. These results were significant (*t*(31) = 2.24, *p* = .03, 95% CI [0.33, 7.21]), indicating a reduction in symptom severity (i.e., post-intervention SPQ scores were lower than pre-intervention scores), suggesting the intervention might have influenced participant fear of needles. A paired t-test examining life interference at baseline and at one-month follow-up was not significant (*t*(31) = –0.15, *p* = .88, 95% CI [-3.21, 2.78]).

To further evaluate the possible effects of this single session of VRET on fear of needles, participants’ anxiety levels after encountering images of needles and injections were compared between groups. Before viewing the photos at baseline, anxiety levels did not differ between the groups (*W* = 565.00, *p* = .23, *r* = 0.15). However, after viewing the same photos of needles and injections, the experimental group reported significantly lower anxiety levels compared to the comparison group (*W* = 695.00, *p* = .002, *r* = 0.39). Furthermore, the reduction in anxiety from pre- to post-exposure was significantly greater in the experimental group, indicating a moderate effect of the intervention (*W* = 684.00, *p* = .004, *r* = 0.37).

To independently assess the magnitude of change in symptom severity, life interference, and affect from baseline to one-month follow-up, a series of standalone effect size calculations were conducted. A small effect size indicated a difference in symptom severity between baseline and one-month follow-up contamination scores of *g* = 0.23 for the MFS and *g* = 0.35 for the SPQ. Effect size was negligible (*g* = 0.009) for life interference. For negative affect, a small negative effect size (*g* = –0.35) was found, while a large positive effect size (*g* = 0.87) was found for positive affect. This suggests a small decrease in negative affect and a large increase in positive affect.

### User experience

To explore how participants in the experimental group perceived the virtual environment, VR presence (i.e., feeling physically present and engaged in the VR environment) and plausibility were investigated.

**Figure 6:**
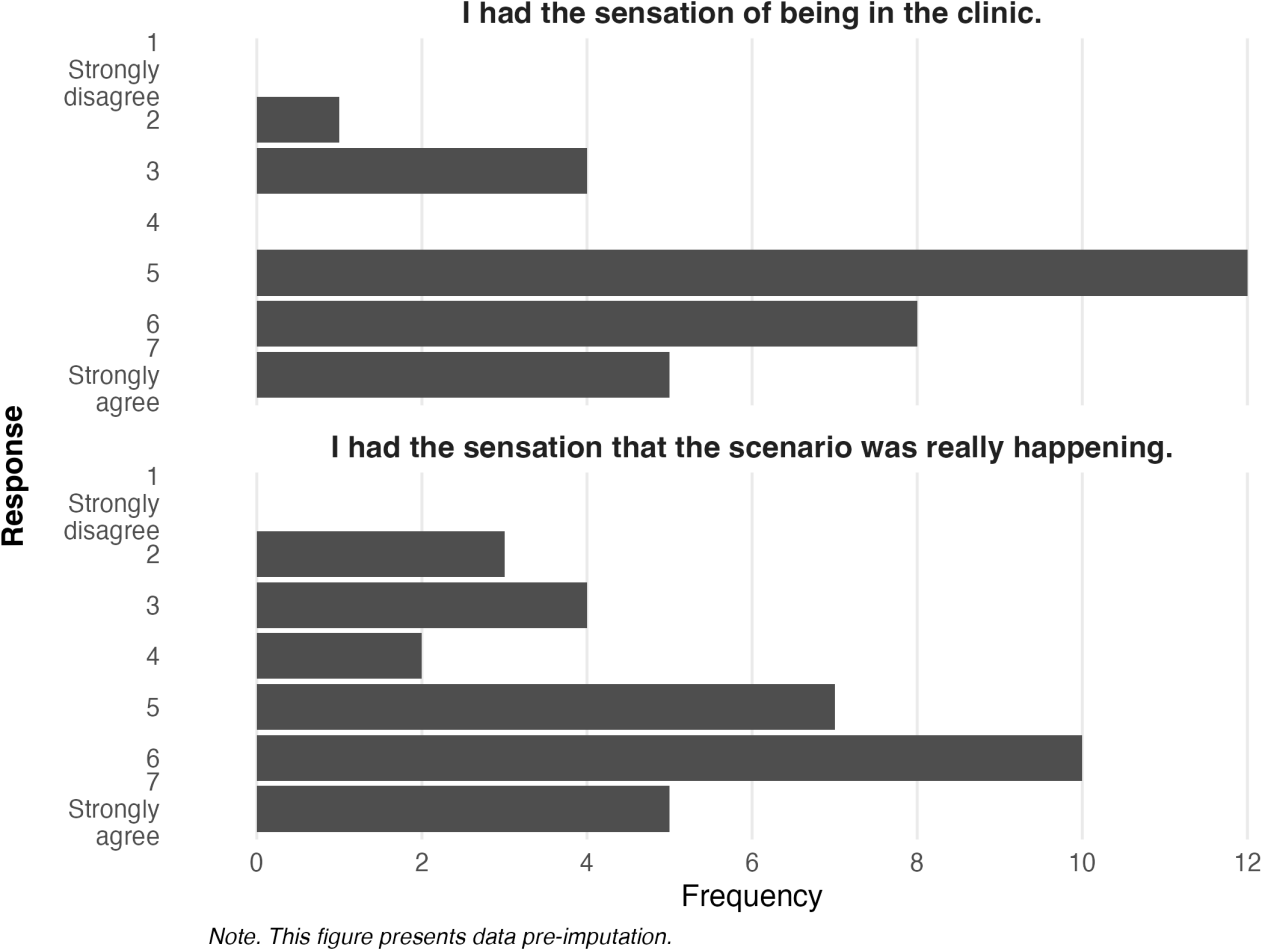
Frequencies of endorsement for presence in the virtual environment.

**Figure 6** explores participant responses for questions related to virtual presence. Responses indicate that the majority of participants who experienced the VRET intervention believed it captured the sensation of being in a medical clinic (*M* = 5.22, *SD* = 1.27) and that the scenario was really happening (*M* = 5.00, *SD* = 1.54).

**Figure 7:**
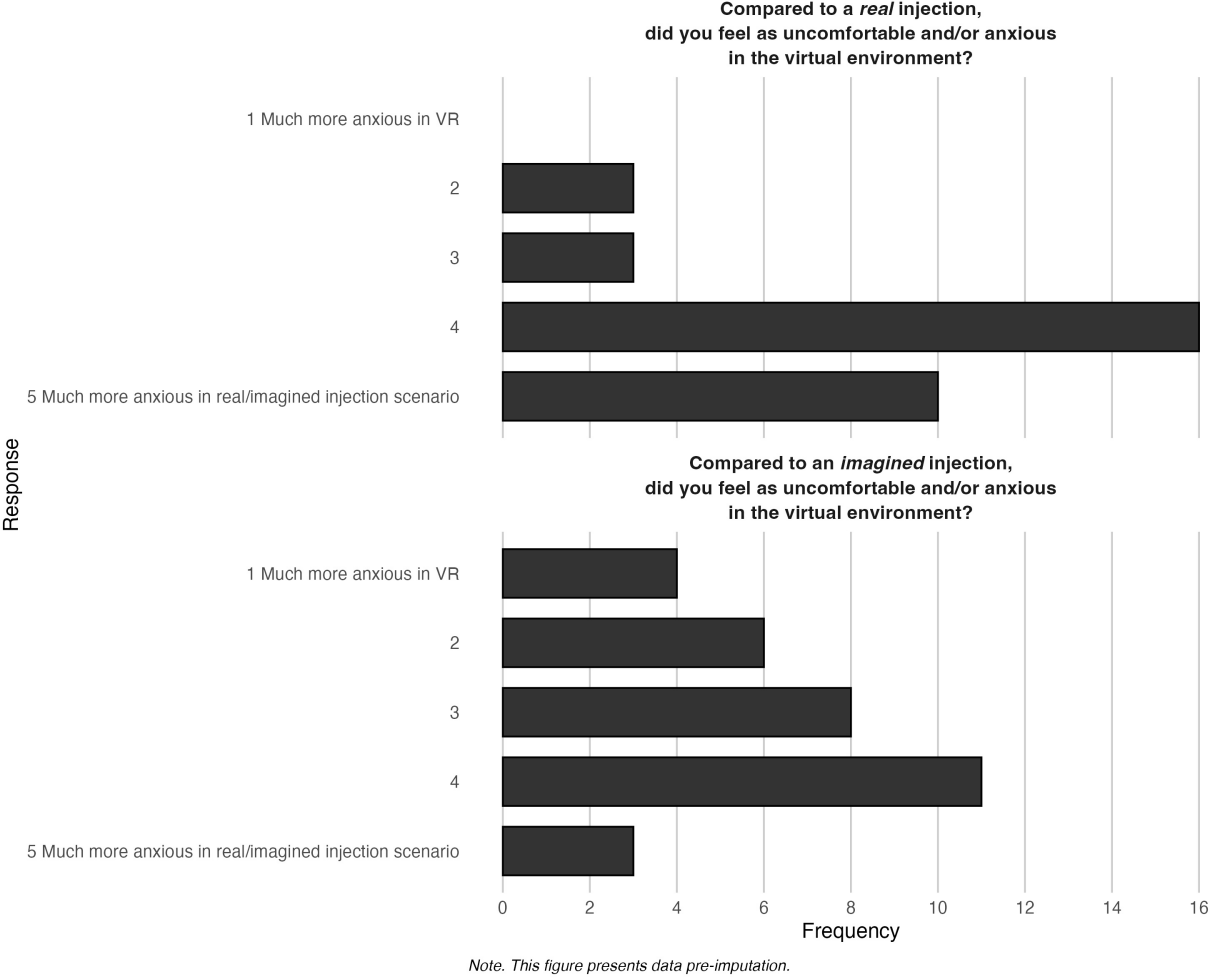
Frequencies of endorsement for plausibility in the virtual environment.

**Figure 7** displays the findings related to plausibility in the virtual environment. There was a varied response from participants; when compared to a real (*M* = 4.03, *SD* = 0.88) and an imagined scenario (*M* = 3.09, *SD* = 1.18), some found the virtual environment anxiety-provoking, while others did not.

Participants reported high levels of usability for the VRET intervention, with average scores of 73.36 (*Mdn* = 75.00, *SD* = 17.29) on the SUS, where a score of 68 is generally considered average and acceptable (36, 42). Many participants reported that they would like to use the VR system frequently (*M* = 3.19, *SD* = 1.07). They thought it was easy to use (*M* = 4.00, *SD* = 1.12), would not need the support of a technical person to be able to use it (reverse coded: *M* = 1.97, *SD* = 0.85), believed that most people would learn to use it very quickly (*M* = 3.91, *SD* = 0.80), and felt very confident using it (*M* = 3.75, *SD* = 1.03). Additionally, they did not find the system unnecessarily complex (reverse coded: *M* = 1.59, *SD* = 0.96).

In the open-ended questions, a participant described using the intervention as “…a really good idea and helps rehearse the scenario beforehand so it feels a lot less anxiety inducing. It felt quite real.” Another participant wrote, “I actually think that if I did this periodically over a few months and especially right before an injection, it could help my phobia. I think my fear is heightened because I don’t often have injections, so my anxiety is higher when I have them because I am not used to them … This experience could help me get used to the process and seeing needles, which would hopefully make me calmer in that situation in real life. If there are needles on tv/in films I always look away but this has made me think my phobia could improve if I expose myself to them more, as they will become more normal.” Complete participant feedback is listed in **Supplementary Table S2** in the Appendix.

7 participants reported VR sickness at the end of the intervention. **Table 5** examines VR sickness scores as measured by the SSQ at baseline and after the intervention. Disorientation was the highest reported symptom. Total VR sickness was substantially higher after the VRET intervention (*M* = 101.23, *Mdn* = 93.50) than at baseline before any VR engagement (*M* = 19.95, *Mdn* = 11.22). Total score for VR sickness ranged from 0 to 71.06 at baseline and 78.54 to 175.78 at the end of the intervention.

**Table 5:** Simulator sickness questionnaire scores at baseline and end of intervention.

Time point	Nausea	Oculomotor	Disorientation	Total score
	M, Mdn(SD)	M, Mdn(SD)	M, Mdn(SD)	M, Mdn(SD)
Baseline	14.46, 9.54 (14.12)	18.95, 11.37 (20.57)	19.76, 0.00 (33.48)	19.95, 11.22 (23.02)
End of Intervention	83.09, 76.32 (15.02)	72.77, 68.22 (18.20)	123.93, 111.36 (35.83)	101.23, 93.50 (21.54)

Participants suggested several areas of improvement to the VRET that might increase their feeling of engagement and immersion in the experience, such as the graphical representation of people in the environment. Some highlighted wanting more pressure or a pinch on their arm as their avatar received the injection in the VR environment to simulate the pain of receiving an injection. Participants also mentioned adding “sounds that you’d normally hear in a hospital [other than] conversations between people” and “more happening around me as it would be in a real clinic.” **Supplementary Table S2** in the Appendix includes a full list of participant feedback.

## Discussion

This study aimed to build on previous literature to evaluate the efficacy and acceptability of VRET for fear of needles in adults. Previous research on this topic has concentrated on specific subsets of various populations rather than adults more generally (e.g., 16, 17). Our study extends prior VRET work by specifically targeting fear of needles in adults with a range of severity of symptoms and examines user experience and acceptability as a central aim.

### Efficacy of the intervention

The results suggest that VRET can effectively elicit anxiety during exposure while also being highly acceptable to participants. As expected, participants reported high levels of anxiety during virtual exposure to needle-related stimuli, supporting the validity of VRET as an exposure tool. The first VR exposure elicited higher average levels of anxiety compared to the second exposure, likely due to anticipatory anxiety and the novelty of the experience in the beginning. In the second VR exposure, participants demonstrated some habituation and were more familiar both with the environment and the protocol.

In addition to reductions in anxiety, we observed modest increases in positive affect and decreases in negative affect following the VR exposures. Although these changes could partially reflect participants’ relief that the exposures had ended, such shifts may also serve as markers of emotional recovery and regulatory processing. Theoretical models of exposure therapy informed by inhibitory learning provide a conceptual framework for understanding this: effective emotion regulation and positive affect following exposure may enhance consolidation of new safety memories and reduce fear return, whereas poor affective recovery may undermine long-term outcomes (34, 43). These findings are also consistent with previous research reporting similar affective patterns following virtual exposure (32, 33). Thus, tracking positive and negative affect offers insight into participants’ emotional coping and immediate intervention impact.

However, the findings regarding symptom reduction were more nuanced. While fear severity scores decreased significantly from baseline to post-intervention according to the SPQ, no significant change was found using the MFS over this period of time. This discrepancy suggests that different measures of fear might capture distinct aspects of the phobic response, highlighting the need for further research into the most appropriate outcome measures for VRET interventions.

While the immediate reductions in fear-related symptoms were promising, the findings indicate that a single session may not be sufficient for long-term change. Instead, repeated exposure may be required to ensure lasting effects. One explanation for this is that a single session might create only a transient decrease in anxiety, whereas more extensive treatment protocols may be required to achieve lasting change. While a single session of exposure therapy can temporarily reduce fear, its effects may be short-lived because deep-seated fear responses often necessitate repeated exposure for lasting change. The mixed results indicate that a single session might not provide sufficient reinforcement to counteract these ingrained responses, underscoring the significance of multiple sessions to strengthen and solidify the benefits over time. However, further research is needed to determine the optimal number of sessions required to fully eliminate fear of needles.

Nevertheless, the current results provide compelling evidence that even a single session of VRET can effectively reduce anxiety related to needle phobia. The significant reduction in anxiety and increase in positive affect observed in the experimental group following the intervention suggests that VRET is a promising tool for addressing fear responses in clinical and subclinical populations. Notably, the comparison group, which did not receive the intervention, showed no significant changes in affect or anxiety, reinforcing the specificity of the VRET effect. Furthermore, the between-group analysis revealed that the experimental group experienced significantly lower anxiety after viewing needle-related images compared to the comparison group, despite no initial difference at baseline. This post-intervention contrast, alongside the greater reduction in anxiety within the experimental group, underscores the potential of VRET to buffer individuals against anxiety responses triggered by phobic stimuli. Taken together, these findings support the efficacy of brief, targeted VRET interventions in modulating both emotional reactivity and affective states, particularly in the context of needle-related fears.

### User experience

A pivotal aim of this study was assessing the user experience and acceptability of VRET for adults with fear of needles. The results indicate that participants found the VR environment to be immersive and engaging, with many reporting a strong sense of presence and realism in the virtual clinic. The majority of participants endorsed high levels of plausibility, suggesting that the virtual scenario was sufficiently realistic to elicit meaningful emotional and physiological responses. This supports prior findings that VRET can effectively simulate real-world anxiety inducing situations while maintaining a controlled and safe environment for participants (e.g., 11, 18). Additionally, participants reported high usability scores on the SUS. Most indicated they found the VR system easy to use, felt confident using it and did not need to learn a lot of things beforehand.

Our findings suggest that the VR intervention was generally well received by participants in terms of both usability and broader experiential acceptability. Although the SUS does not directly assess acceptability, its use as a proxy for usability-related acceptability is consistent with digital health literature and the Theoretical Framework of Acceptability (40). Importantly, our interpretation of acceptability extended beyond SUS scores alone. Open-ended feedback captured participants’ affective and cognitive responses, such as feelings of emotional engagement and willingness to use the intervention again. These qualitative data provided valuable context for understanding the overall acceptability of the intervention as a user-centered therapeutic tool.

Beyond usability and acceptability, we also examined participants’ physical comfort and any adverse reactions associated with the VR experience. In interpreting the VR sickness findings, it is important to consider the potential overlap between symptoms captured by the SSQ and those that may arise from fear of needles. Physiological responses to fear, such as dizziness, nausea, and light-headedness, can resemble symptoms typically associated with VR sickness. As such, elevated SSQ scores in this context may reflect, in part, participants’ needle-related distress rather than purely VR-induced discomfort. While this possibility does not undermine the utility of the SSQ, it does suggest that scores should be interpreted cautiously, particularly in phobia contexts where symptom overlap is likely. Future studies might benefit from collecting additional physiological or subjective data to help disambiguate the source of these symptoms.

The usability and acceptability of VRET underscores its feasibility as an alternative to traditional exposure therapy, particularly for individuals with difficulty accessing in-person treatment. This is in line with previous literature (44), which found a significantly lower rate of refusal for VRET compared to *in vivo* exposure. Open-ended feedback further emphasized the intervention’s potential for reducing fear over time, with several participants suggesting that they believed repeated exposure to the virtual scenarios could help normalize needle-related experiences. Some participants also noted the potential for VR sickness, suggesting that improvements in VR technology and session structuring may enhance user comfort.

### Limitations

This study has several limitations that affect its generalizability. First, the small sample size requires replication with a larger, more diverse sample. Second, the comparison group was recruited via Prolific, rather than using a control group that was randomized and recruited in the same manner as the experimental group. This difference introduces potential limitations in comparability. Nevertheless, individuals in the comparison group were asked if they would be willing to participate in a VR-delivered intervention targeting fear of needles. Those who declined were excluded from the group, enhancing comparability. Additionally, clinical cut-off scores for the SPQ and MFS are not formally established, and both instruments are typically interpreted using sample-specific thresholds. However, both the SPQ and MFS are validated, psychometrically sound instruments that are widely used in clinical and experimental research on specific phobias, including fear of needles. In the present study, scores were interpreted descriptively and used primarily to assess relative change and within-sample comparisons, rather than to make diagnostic determinations.

Despite limitations, this study offers several strengths. First, it addresses a critical gap in the literature by specifically examining VRET for fear of needles, rather than general blood-injection-injury phobia. Second, by focusing on a community sample of adults rather than narrower adult or pediatric populations, the study offers broader insights into needle fear across a diverse age range. Third, the study’s central aim of examining user experience and acceptability alongside efficacy provides valuable insights into the subjective experience of VRET, offering guidance for refining future VR-based interventions. Future studies should consider in-person control groups to enhance methodological rigor. Lastly, while this study focused on a single-session VRET intervention, the effects of multi-session protocols over the course of multiple days remain largely unexplored and have strong potential for more robust effects.

## Conclusion

This study contributes to the growing body of research on VRET as a potential intervention for fear of needles in adults. The promising findings suggest that VRET can effectively elicit anxiety during exposure, while simultaneously being highly acceptable to participants. Our results also suggest that VRET has the potential to improve treatment accessibility for individuals with fear of needles and injections, with significant implications for public health. Given the importance of medical procedures involving needles, from vaccinations to blood tests, interventions that help individuals manage their fears are highly valuable. If validated in future larger-scale studies, VRET could serve as a scalable and accessible treatment for individuals who fear needles, ultimately improving medical adherence and health outcomes.

## Data Availability

The raw data supporting the conclusions of this article will be made available by the authors, without undue reservation.
