# Supplementary material for: Virtual reality delivered exposure for fear of needles: a small-scale pilot
Source: Front Psychiatry. 2025 Sep 11;16:1642988. doi: 10.3389/fpsyt.2025.1642988 (PMC12462493; doi:10.3389/fpsyt.2025.1642988)
Supplement: Supplementary file 1 [file DataSheet1.pdf]

Table S1

Shapiro-Wilk Test Results for Main Study Variables

**Experimental Group**		
**Variable**	**W**	**p-value**
MFS Initial Score	0.97	0.545
SPQ Initial Severity Score	0.95	0.198
SPQ Initial Interference Score	0.91	0.011*
Anxiety Pre-Images	0.83	< .001***
Anxiety Post-Images	0.92	0.016*
Anxiety Follow-Up Pre-Image	0.95	0.116
Anxiety Follow-Up Post-Image	0.93	0.035*
Positive Affect at baseline	0.98	0.859
Negative Affect at baseline	0.91	0.011*
Positive Affect at end of intervention	0.88	0.002**
Negative Affect at end of intervention	0.86	0.001**
SPQ Follow-Up Severity Score	0.98	0.799
SPQ Follow-Up Interference Score	0.91	0.015*
MFS Follow-Up Score	0.95	0.199
**Comparison Group**		
**Variable**	**W**	**p-value**
MFS Initial Score	0.85	< .001***
SPQ Initial Severity Score	0.91	0.011*
SPQ Initial Interference Score	0.85	< .001***
Anxiety Pre-Image	0.86	0.001***
Anxiety Post-Image	0.86	0.001***
Positive Affect at baseline	0.96	0.242
Negative Affect at baseline	0.72	< .001***
Positive Affect at end of intervention	0.96	0.282
Negative Affect at end of intervention	0.74	< .001***

Note. $*p < 0.05$. $**p < 0.01$. $***p < 0.001$.

Table S2

Selection of feedback and acceptability open-ended responses

Question	Participant Response
**How did you feel while you were taking part in the virtual reality experience?**	A bit nervous as the VR is quite real, I would say the second time is better as I'm more used to the environment and know what is exactly going to happen.
	The VR experience felt somewhat real, as there was the actual sensation of having an injection/blood taken, albeit on a lesser scale. The thought of actually having blood taken did make me feel quite anxious, however knowing that it wasn't actually happening made me feel somewhat calmer.
	the experience was really interesting. Although the graphics made it clear that it was a simulated environment, certain elements made it feel very real. I felt similarly to how I feel when I am getting an injection.
	I felt interested at first, curious and then a bit more nervous as it looked quite realistic.
	I felt almost exactly as if I were having a real injection/blood test. My palms became quite sweaty and I could feel my heart in my chest. I also felt slightly light headed. It was very realistic, especially being touched at the time the needle goes in.
	Anxious, but more comfortable than in reality. I think it was close enough to reality that I felt anxious, but distant enough that I didn't totally panic.
	I was comfortable overall but felt that the VR scene was quite realistic. I imagined that I was in the same situation and think that my response to the VR and to a real-life scenario would be similar.
	The VR looks almost real. I almost felt like I was in a hospital attending to a nurse.
	The virtual reality was a good insight into replicating the feelings felt when receiving an injection, it reminded me of the same feelings I felt when getting a real injection.
	it was really fun experience and the interaction were pleasant
	Stress of being in an environment that was related to pain.
	overall I was more nervous than I thought, I think the multiple shots actually induced a fear of needles but by the second time it was slightly less scary as I'd done it so many times. the VR itself was quite motion sickness inducing, when he walked towards the clinic.
	The experience felt very real in terms of sensations than visuals. When the blood drawing simulations ended, I felt slight pain in the area of my arm where tactile sensations were presented.

Continued on next page

Table S2 – *Continued from previous page*

Question	Participant Response
	At first, I found the procedures very unexpected and definitely wasn't expecting the experimenter to actually touch my arm to make me feel like I was getting an actual injection. After watching the video and knowing that the same procedure is going to be repeated, I was less anxious.
	felt as if i was really in the clinic and was excited but intrigued.
	It was interesting, the graphics were a little unusual and confusing but it felt fine.
What did you think when you saw the medical environment?	I don't like that, especially the blood samples on the right.
	It felt quite realistic. People seemed serious.
	Curious and uncomfortable but mainly thinking about what might happen/didn't feel very present.
	As I have diabetes, seeing the medical environment didn't phase me much, as this is something that I am quite used to, now.
	Once I knew I was there for an injection I was less willing to be there. Quite realistic, though there weren't the smells I tend to associate with a medical environment.
	It made me feel a bit apprehensive and reminded me of unpleasant situations I have had in hospitals or while visiting my doctor.
	It looked too dissimilar from real life for me to be immersed and actually believe I was in the real situation.
	It looked realistic and I did have the sensation of waiting in a waiting room. Of course the graphics aren't photo real but it still felt as if I was there.
	Slightly nervous but not very anxious because I did not see silver, medical instruments
	Nervous, it looked like a stereotypical medical office with posters about vaccination which always make you anxious anyway!
	Didn't feel like it was very realistic - the fact that it was animated figures definitely made it less nerve wracking that it would normally be for me.
	I thought it was somewhat realistic.
	I felt that it was realistic and showed waiting in a clinic quite well. It put me in the scene and allowed me to imagine that I was really there.

Continued on next page

Table S2 – *Continued from previous page*

Question	Participant Response
	Felt like other medical environments I've been in, just more unfamiliar.
	The setting and environment was very realistic down to the sounds when waiting and how when entering the room to get the injection it is silent and awkward waiting for the injection.
	Felt like I was there for real, especially with the sound coming from different directions.
How did you feel when you saw the needle?	I feel extremely nervous when I saw that, feeling it's going to be injected my skin soon.
	Fear/wanting to pull back, It felt quite 'real', and even more 'real' if I was looking at the needle while it was in.
	I tried to look away when I knew that the needle was 'on show', so that I didn't make myself feel more anxious, from looking at it.
	I tried not to look at the needle. But on the second round I look at it during the longer draw and had to immediately look away.
	Nervous. My hands were sweatier. Less nervous than I would be in reality - I normally can't even look at one going into me.
	It reminded me of a few accidents that I have had while a nurse has tried to take my blood from my vein. There was one time when she took 4 tries before managing it and I remembered it and I felt extremely uneasy.
	I felt anxious and nervous when I saw the needle. During the first session I turned my head away (as I would in real life) but in the second session I forced myself to look at it and it did help seeing the progress of the blood test.
	Irrational, afraid and anxious however I can look at a needle and observe it for a few moments I cannot however, fathom seeing the needle inserted in to my arm.
	First time - scared. After that, not as scared.
	I felt neutral just seeing the needle, rather it was the needle entering my avatar's arm that caused me discomfort. I did not like seeing the needle in the avatar's arm and felt squeamish when I looked at it.
	I felt scared . It reminded me of last time I had to take an injection for vaccine.
	Anxious, nervous and definitely an increased heart rate.

Continued on next page

Table S2 – *Continued from previous page*

**Question**	**Participant Response**
	Anxious, but watching it come near helps.<br><br>not as anxious as I would in real life, when I felt the sensation in real life that was when I actually became nervous.<br><br>I tried to look at the needle in the second part of the experiment, and it still made me uncomfortable. I never look at needles piercing through the skin, so that was a big step for me.<br><br>Anxious, but kind of fun after the first one.
In your opinion, is there anything that could have been done differently to improve the software or create a better experience (in any aspect)?	Pinch the skin for the sensation to feel more realistic.<br><br>The nurse could have been a lot more friendly and less serious.<br><br>The movement/design of the VR could be more realistic, to help keep more similarities between the VR environment, and 'real life'.<br><br>The graphics were the primary thing that made it difficult to fully engage/become immersed in the experience. Perhaps changing the dialogue a little bit in the second round, and some more context behind the injections.<br><br>To make it more realistic- maybe more sounds that you'd normally hear in a hospital (less focused on conversations between people).<br><br>The eye level of the avatar could possibly be altered to each subject. I could not immerse myself in the simulation especially because I did not feel like I was the avatar getting the injection.<br><br>Just the bit where you walk into the doctor's office was strange as I was sitting down but my VR self was walking so it made me a bit motion sick. If the graphics of the people could improve that would help too. But in general I thought it was very good.<br><br>I'm not sure- maybe something to do with some background about why I needed the injection/blood draw etc. maybe it would increase the feeling of obligation which would usually motivate me getting an injection.<br><br>Maybe consider adding headphones to the set up to enhance the hearing effects.<br><br>Maybe have real life people in the VR instead of 3D characters.

Continued on next page

Table S2 – *Continued from previous page*

Question	Participant Response
	Making me stand up and walk around when my avatar was told to, and telling me to extend my arm to do the tests when my avatar did.
	To improve (this would be hard) but replicating the pain when getting the needle injected would make the setting be more realistic, though requiring consent, anything pointy and putting more pressure would make it more realistic.
	More happening around me as it would be in a real clinic.
	The human models could move their lips as they speak and the models could be more rendered, making them more realistic.
	The characters and door closing need to be more realistic.
	The color gradient wasn't quite accurate and the clothing particularly as the characters moved it looked rather unnatural.
Do you have any additional comments about the virtual reality program and/or your experience with it?	It was an enjoyable, fun experience and a good way for exposure therapy without causing too much distress.
	I think the VR is quite real, it has both images, sounds
	conversations to make it more realistic. Perhaps can simulate the hospital smell too.
	It was very fun and interesting, sitting in the clinic waiting area felt real but going inside the room for injection not so much.
	I think its a really good idea and helps rehearse the scenario beforehand so it feels a lot less anxiety inducing. It felt quite real.
	It was quite odd, and I wasn't expecting it to be as life like as it was even though it was animated.
	The movement of the people in the environment was not 'true to life', and therefore reminded me that this was a virtual experience, and the fact that the experience was more cartoon-y made me remember that this wasn't real.
	I think that the program is a good idea, and if developed more, could have applications beyond simply study. I think that injections are a good application for this.
	it was quite realistic in simulating the scenario but not so much at the same time. i could remind myself it wasn't real, but it was very comfortable to use and look around with.
	It was a very interesting experience and I think it simulates a real environment quite well.

Continued on next page

Table S2 – *Continued from previous page*

**Question**	**Participant Response**
	I think the virtual program could use real people instead of cartoon characters to help individuals truly get immersed in the simulation.<br><br>I actually think that if I did this periodically over a few months and especially right before an injection, it could help my phobia. I think my fear is heightened because I don't often have injections, so my anxiety is higher when I have them because I am not used to them. Whereas this experience could help me get used to the process and seeing needles, which would hopefully make me calmer in that situation in real life. If there are needles on tv/in films I always look away but this has made me think my phobia could improve if I expose myself to them more as I they will become more normal.<br><br>The character models and movements can be improved with motion capture technology for more realistic display.<br><br>Using VR is really good to immerse people in a desired environment, replicating the sounds and settings even up to the skin color of the person.<br><br>More immersion in acting out what was asked, like squeezing your hands, putting your arm out etc may have helped remove disassociation.<br><br>It was more realistic than I'd expected, but please change the part where he walks, it made me quite nauseous. everything else was great and really simulated the anxiety of getting a shot at the GP.<br><br>I think it really helped with needle nerves but I noticed some of the people in the waiting room their eyes weren't animated properly , kind of skinwalker eyes. was uncanny/eerie.
